# Editorial: Unlocking the secrets to healthy brain aging: diet, nutritional bioactive compounds, and lifestyle

**DOI:** 10.3389/fnut.2026.1995444

**Published:** 2026-10-05

**Authors:** Cristina Angeloni, Daniel Di Risola, Roberto Mattioli, Luciana Mosca

**Affiliations:** 1 Department for Life Quality Studies, Alma Mater Studiorum, University of Bologna, Rimini, Italy; 2 Department of Biochemical Sciences “A. Rossi Fanelli”, Sapienza University of Rome, Rome, Italy; 3 Laboratory of Biochemistry and Molecular Biology, Department of Movement, Human and Health Sciences, Università degli Studi di Roma “Foro Italico”, Rome, Italy; 4 Center for Research in Neurobiology ‘Daniel Bovet' (CRiN), Sapienza University of Rome, Rome, Italy

**Keywords:** bioactive compounds, cognitive performance, diet, healthy brain aging, lifestyle, neurodegeneration, polyphenols, vitamins

In recent years, research on “healthy brain aging” has highlighted the existence of an extraordinarily complex interplay among the brain, diet, nutritional bioactive compounds, and lifestyle factors. Within this context, the field is undergoing a major conceptual shift. Traditionally, research has primarily focused on identifying individual nutrients or isolated factors, essentially single variables, capable of preventing cognitive decline and preserving the health of the brain and nervous system in the broadest sense. Today, however, the importance of a systems-level perspective is becoming increasingly evident, whereby foods, their bioactive constituents, and lifestyle habits act as modulators of complex biological networks, contrasting with the targeted approach of a single drug directed at a specific pathway or enzyme.

Supporting this conceptual transition, Meng et al., conducted a bibliometric analysis spanning more than three decades of research on nutrition and Alzheimer's disease (AD), one of the most extensively studied neurological disorders. Their findings revealed a progressive shift in scientific focus from individual nutrients and classical pathogenic mechanisms toward a more integrated framework in which diet, metabolism, lifestyle, and individual susceptibility are regarded as interdependent elements. Also, migration experience, which may be associated with higher educational attainment, greater social engagement, and increased cognitive stimulation, have also been linked to better brain health (Takahashi et al.). From a nutritional point of view, the interactions among multiple bioactive molecules and both intrinsic and extrinsic factors may contribute to the limited efficacy observed in certain nutritional interventions in clinical trials (Chiriac et al.) as well as the considerable heterogeneity reported across the scientific literature. A representative example is provided by clinical studies evaluating supplementation with individual vitamins to prevent the onset or progression of AD. Many of these investigations

have yielded conflicting results (Miteva et al.). Although the association between AD and hypovitaminosis appears convincing, it remains unclear whether a causal relationship exists. Specifically, it is uncertain whether vitamin deficiency contributes to disease development and progression or whether the disease itself induces vitamin deficiencies through alterations in appetite and dietary habits. At the same time, while supplementation with individual vitamins appears unable to effectively counteract disease progression, combinations of multiple vitamins have been reported to improve lipid peroxidation, brain atrophy, and several AD-related biomarkers, including homocysteine, amyloid-β (Aβ), and tumor necrosis factor-α (TNF-α). Particularly illustrative is the case of vitamin E, whose antioxidant activity depends on regeneration by vitamin C; in the absence of adequate levels of vitamin C, vitamin E itself may acquire pro-oxidant properties. This example clearly demonstrates that biochemical context is a critical determinant of the effectiveness of a nutritional intervention.

Another important aspect emerging from recent studies concerns the relationship between dose and biological effect. Traditionally, it has been assumed that higher nutrient intake inevitably results in greater biological benefit. However, this assumption may not always hold true. For example, He and colleagues (He et al.), through an observational study, identified a non-linear association between dietary fiber intake and cognitive function, showing that cognitive performance improves with increasing fiber consumption only up to a certain threshold, beyond which additional intake produces little or no further benefit. This finding introduces a fundamental concept: many nutrients are likely characterized by an optimal intake window, whereas excessive doses may not only fail to provide additional benefits but could even become counterproductive. Even more intriguing is the observation that much of the association between dietary fiber intake and cognitive function appears to be mediated by vitamin E.

The synergy between molecules represents another important pharmacological tool in brain health and should not be overlooked. Examples of synergic activity among similar molecules were presented in the issue by two different research groups. The combined administration of marine-derived polyunsaturated fatty acids (PUFAs) and bioactive peptides exemplifies how the efficacy of bioactive compounds depends not only on their intrinsic properties but also on their ability to simultaneously modulate multiple pathophysiological processes. In the case of marine omega-3 fatty acids, docosahexaenoic acid (DHA) and eicosapentaenoic acid (EPA) influence neuronal membrane fluidity, synaptic function, and neuroinflammation, whereas marine bioactive peptides exert antioxidant, anti-apoptotic, and neuroprotective activities (Yang et al.). These compounds should therefore be viewed not as alternatives but as complementary agents, whose combined administration may produce synergistic or additive neuroprotective effects, exceeding those expected from exceeding those expected from single-molecule supplementation.

This concept can also be extended to other bioactive compounds, such as polyphenols. Specifically, the biological efficacy of polyphenols arises not only from the synergistic interaction among diverse bioactive molecules, but also from the pleiotropic nature of individual polyphenols, which can influence numerous biological pathways through distinct mechanisms of action. In the treatment of major depressive disorder, for example, polyphenols appear to act on multiple molecular targets through different mechanisms, simultaneously modulating neuroinflammation, oxidative stress, mitochondrial function, neurogenesis, neurotransmission, as well as the gut microbiota and gut-brain axis (Zhu et al.). Rather than acting on a single specific pathway, these compounds seem to promote the restoration of homeostasis within a dysregulated biological system, consistent with the modern concept of network pharmacology.

If brain health depends on the interaction of all these variables, then nutritional interventions, including oral supplementation, cannot follow a one-size-fits-all approach. Instead, they should be tailored to the characteristics of each individual, including age, sex, food intolerances, personal preferences, cultural background, and other relevant factors. The success of a nutritional intervention therefore depends not only on the composition of the supplement itself but also on the ability to personalize treatment according to the clinical context and individual characteristics. From this perspective, experimental models may also need to evolve toward more pragmatic approaches, adopting, where appropriate, “preference-adaptive” study designs that may better reflect real world clinical practice than the conventional designs employed in most randomized trials (Feng et al.).

Taken together, the studies published in this Research Topic outline a profound transformation in nutritional research, shifting the emphasis from the search for a “miracle product” toward understanding the biological networks that regulate brain aging. Although these studies address seemingly diverse topics, from polyunsaturated fatty acids and polyphenols to vitamins and dietary fiber, they converge on a common framework centered on precision nutrition and the maintenance of brain resilience during aging. Overall, the contributions point toward a future in which nutritional interventions are increasingly personalized, based on combinations of bioactive compounds, optimized dosing strategies, and comprehensive characterization of each individual's biological profile. The effectiveness of a nutritional strategy will likely depend on integrating specific nutrients within a complex biological system that encompasses genetic background, metabolic status, gut microbiota, lifestyle, and the stage of the aging process.

